# Supplementary material for: Pyruvate Dehydrogenase Kinase 1 inhibition mediated oxidative phosphorylation enhancement in cartilage promotes osteoarthritis progression
Source: BMC Musculoskelet Disord. 2023 Jul 20;24:597. doi: 10.1186/s12891-023-06585-6 (PMC10357736; doi:10.1186/s12891-023-06585-6)
Supplement: Supplementary file 1 — Additional file 1. [file 12891_2023_6585_MOESM1_ESM.zip › MF.Gsea.1653627316242/GOMF_PROTEIN_SERINE_THREONINE_KINASE_ACTIVATOR_ACTIVITY.html]

Details for gene set GOMF\_PROTEIN\_SERINE\_THREONINE\_KINASE\_ACTIVATOR\_ACTIVITY[GSEA]

|  || Dataset | 55457\_collapsed\_to\_symbols.cls.cls #OA\_versus\_normal.cls.cls #OA\_versus\_normal\_repos |
| Phenotype | cls.cls#OA\_versus\_normal\_repos |
| Upregulated in class | normal |
| GeneSet | GOMF\_PROTEIN\_SERINE\_THREONINE\_KINASE\_ACTIVATOR\_ACTIVITY |
| Enrichment Score (ES) | -0.41427955 |
| Normalized Enrichment Score (NES) | -1.530058 |
| Nominal p-value | 0.018998273 |
| FDR q-value | 0.16573556 |
| FWER p-Value | 1.0 |
Table: GSEA Results Summary

  

Fig 1: Enrichment plot: GOMF\_PROTEIN\_SERINE\_THREONINE\_KINASE\_ACTIVATOR\_ACTIVITY      
 Profile of the Running ES Score & Positions of GeneSet Members on the Rank Ordered List

  

| SYMBOL | TITLE | RANK IN GENE LIST | RANK METRIC SCORE | RUNNING ES | CORE ENRICHMENT || 1 | CKS1B | CDC28 protein kinase regulatory subunit 1B [Source:HGNC Symbol;Acc:HGNC:19083] | 927 | 0.533 | -0.0331 | No |
| 2 | CALM3 | calmodulin 3 [Source:HGNC Symbol;Acc:HGNC:1449] | 1039 | 0.510 | -0.0045 | No |
| 3 | PARP16 | poly(ADP-ribose) polymerase family member 16 [Source:HGNC Symbol;Acc:HGNC:26040] | 1997 | 0.361 | -0.0526 | No |
| 4 | MLST8 | "MTOR associated protein, LST8 homolog [Source:HGNC Symbol;Acc:HGNC:24825]" | 2259 | 0.331 | -0.0487 | No |
| 5 | CCNB1 | cyclin B1 [Source:HGNC Symbol;Acc:HGNC:1579] | 2359 | 0.319 | -0.0331 | No |
| 6 | ETAA1 | ETAA1 activator of ATR kinase [Source:HGNC Symbol;Acc:HGNC:24648] | 2707 | 0.279 | -0.0397 | No |
| 7 | BMPR1A | bone morphogenetic protein receptor type 1A [Source:HGNC Symbol;Acc:HGNC:1076] | 2745 | 0.275 | -0.0224 | No |
| 8 | DAZAP2 | DAZ associated protein 2 [Source:HGNC Symbol;Acc:HGNC:2684] | 3076 | 0.242 | -0.0304 | No |
| 9 | STRADA | STE20 related adaptor alpha [Source:HGNC Symbol;Acc:HGNC:30172] | 3210 | 0.229 | -0.0240 | No |
| 10 | INCENP | inner centromere protein [Source:HGNC Symbol;Acc:HGNC:6058] | 3533 | 0.199 | -0.0345 | No |
| 11 | CAB39 | calcium binding protein 39 [Source:HGNC Symbol;Acc:HGNC:20292] | 4181 | 0.140 | -0.0746 | No |
| 12 | DBF4B | DBF4 zinc finger B [Source:HGNC Symbol;Acc:HGNC:17883] | 4251 | 0.133 | -0.0703 | No |
| 13 | CDK5R2 | cyclin dependent kinase 5 regulatory subunit 2 [Source:HGNC Symbol;Acc:HGNC:1776] | 4273 | 0.131 | -0.0624 | No |
| 14 | IGF2 | insulin like growth factor 2 [Source:HGNC Symbol;Acc:HGNC:5466] | 4378 | 0.123 | -0.0615 | No |
| 15 | LTF | lactotransferrin [Source:HGNC Symbol;Acc:HGNC:6720] | 4452 | 0.117 | -0.0586 | No |
| 16 | MNAT1 | MNAT1 component of CDK activating kinase [Source:HGNC Symbol;Acc:HGNC:7181] | 4681 | 0.098 | -0.0692 | No |
| 17 | CAB39L | calcium binding protein 39 like [Source:HGNC Symbol;Acc:HGNC:20290] | 5286 | 0.049 | -0.1126 | No |
| 18 | CD40LG | CD40 ligand [Source:HGNC Symbol;Acc:HGNC:11935] | 5529 | 0.030 | -0.1293 | No |
| 19 | MAP3K13 | mitogen-activated protein kinase kinase kinase 13 [Source:HGNC Symbol;Acc:HGNC:6852] | 5728 | 0.013 | -0.1437 | No |
| 20 | MTCP1 | mature T cell proliferation 1 [Source:HGNC Symbol;Acc:HGNC:7423] | 5917 | 0.001 | -0.1582 | No |
| 21 | BMPR1B | bone morphogenetic protein receptor type 1B [Source:HGNC Symbol;Acc:HGNC:1077] | 5959 | -0.002 | -0.1613 | No |
| 22 | TCL1A | TCL1 family AKT coactivator A [Source:HGNC Symbol;Acc:HGNC:11648] | 6433 | -0.038 | -0.1953 | No |
| 23 | MAP2K6 | mitogen-activated protein kinase kinase 6 [Source:HGNC Symbol;Acc:HGNC:6846] | 7246 | -0.099 | -0.2512 | No |
| 24 | CCNT2 | cyclin T2 [Source:HGNC Symbol;Acc:HGNC:1600] | 8192 | -0.170 | -0.3123 | No |
| 25 | IQGAP1 | IQ motif containing GTPase activating protein 1 [Source:HGNC Symbol;Acc:HGNC:6110] | 8276 | -0.177 | -0.3058 | No |
| 26 | SPRY2 | sprouty RTK signaling antagonist 2 [Source:HGNC Symbol;Acc:HGNC:11270] | 9531 | -0.277 | -0.3832 | No |
| 27 | MAP2K2 | mitogen-activated protein kinase kinase 2 [Source:HGNC Symbol;Acc:HGNC:6842] | 9932 | -0.315 | -0.3913 | Yes |
| 28 | CALM1 | calmodulin 1 [Source:HGNC Symbol;Acc:HGNC:1442] | 10136 | -0.334 | -0.3826 | Yes |
| 29 | MAP2K1 | mitogen-activated protein kinase kinase 1 [Source:HGNC Symbol;Acc:HGNC:6840] | 10197 | -0.340 | -0.3624 | Yes |
| 30 | CALM2 | calmodulin 2 [Source:HGNC Symbol;Acc:HGNC:1445] | 10579 | -0.382 | -0.3642 | Yes |
| 31 | TCL1B | TCL1 family AKT coactivator B [Source:HGNC Symbol;Acc:HGNC:11649] | 10636 | -0.386 | -0.3403 | Yes |
| 32 | STK3 | serine/threonine kinase 3 [Source:HGNC Symbol;Acc:HGNC:11406] | 10656 | -0.388 | -0.3134 | Yes |
| 33 | CKS2 | CDC28 protein kinase regulatory subunit 2 [Source:HGNC Symbol;Acc:HGNC:2000] | 10695 | -0.392 | -0.2878 | Yes |
| 34 | TAOK2 | TAO kinase 2 [Source:HGNC Symbol;Acc:HGNC:16835] | 10718 | -0.395 | -0.2606 | Yes |
| 35 | BMP2 | bone morphogenetic protein 2 [Source:HGNC Symbol;Acc:HGNC:1069] | 10919 | -0.420 | -0.2455 | Yes |
| 36 | CDK5R1 | cyclin dependent kinase 5 regulatory subunit 1 [Source:HGNC Symbol;Acc:HGNC:1775] | 10985 | -0.428 | -0.2193 | Yes |
| 37 | STK4 | serine/threonine kinase 4 [Source:HGNC Symbol;Acc:HGNC:11408] | 11074 | -0.441 | -0.1939 | Yes |
| 38 | BMPR2 | bone morphogenetic protein receptor type 2 [Source:HGNC Symbol;Acc:HGNC:1078] | 11174 | -0.454 | -0.1685 | Yes |
| 39 | CCNK | cyclin K [Source:HGNC Symbol;Acc:HGNC:1596] | 11330 | -0.475 | -0.1458 | Yes |
| 40 | MAP3K12 | mitogen-activated protein kinase kinase kinase 12 [Source:HGNC Symbol;Acc:HGNC:6851] | 11344 | -0.478 | -0.1119 | Yes |
| 41 | DBF4 | DBF4 zinc finger [Source:HGNC Symbol;Acc:HGNC:17364] | 11855 | -0.561 | -0.1106 | Yes |
| 42 | CCNT1 | cyclin T1 [Source:HGNC Symbol;Acc:HGNC:1599] | 12517 | -0.757 | -0.1068 | Yes |
| 43 | TAOK1 | TAO kinase 1 [Source:HGNC Symbol;Acc:HGNC:29259] | 12753 | -0.918 | -0.0580 | Yes |
| 44 | DDX3X | DEAD-box helicase 3 X-linked [Source:HGNC Symbol;Acc:HGNC:2745] | 12788 | -0.949 | 0.0087 | Yes |
Table: GSEA details [plain text format]

  

Fig 2: GOMF\_PROTEIN\_SERINE\_THREONINE\_KINASE\_ACTIVATOR\_ACTIVITY      
 Blue-Pink O' Gram in the Space of the Analyzed GeneSet

  

Fig 3: GOMF\_PROTEIN\_SERINE\_THREONINE\_KINASE\_ACTIVATOR\_ACTIVITY: Random ES distribution      
 Gene set null distribution of ES for **GOMF\_PROTEIN\_SERINE\_THREONINE\_KINASE\_ACTIVATOR\_ACTIVITY**
